# From intervention studies to national programs, what are the favoring and hindering factors? a scoping review

**DOI:** 10.1186/s12889-025-24770-1

**Published:** 2025-10-28

**Authors:** Renata Cristina Ferreira Rola, Tatiana Rivera Ramirez, Axel Kroeger

**Affiliations:** 1 Centre for Medicine and Society, Faculty of Medicine, University of Freiburg, Freiburg, Germany; 2 Research Group GIGA, University Francisco de Paula Santander, Cucuta, Colombia

**Keywords:** Scaling up, Public health interventions, Translational research, Favoring factors, Hindering factors

## Abstract

**Background:**

The translation of successful health projects into public health practice is among the most relevant challenges to pursue better health results, including the outcomes established by the United Nations Sustainable Development Goals (SDGs). Healthcare interventions proven cost-effective should be expanded into broader policies and practices. This process is called scaling-up and its success depends on several factors. To critically think about how to scale-up projects or adapt programs, it is necessary to identify potential favoring and hindering factors. In this sense, this study aims to document the type of public health programs which have been scaled up, describe the favoring and hindering factors of this process, and critically analyze the findings about it.

**Methods:**

To reach this goal, this project used the scoping review method to map and summarize the existing evidence on scaling up health interventions through available publications. The databases used in this review were PubMed, Web of Science, Cochrane Library, Scopus, VHL, Scielo, and Google Scholar.

**Results:**

Through the selection process from a total of 7,027 search hints, 30 relevant papers were included. The found factors that impacted the scaling-up process were organized based on categories of the ExpandNet/WHO framework. The results indicate that poor “user organization”, “institutional environment” and “resource mobilization” were the most frequently mentioned hindering factors for scaling-up processes. Favoring factors included strong leadership, well defined roles, good coordination, positive political environment and community support and engagement.

**Conclusion:**

Hindering and favoring factors do not exist in isolation, but are correlated, interact, enhance or hinder one another. Beyond the practical advice given in this paper, further research recommended that investigates the association between different factors.

**Supplementary Information:**

The online version contains supplementary material available at 10.1186/s12889-025-24770-1.

## Background

The translation of successful health projects into practice is among the most relevant challenges for achieving better health results in national health programs, including the outcomes established by the United Nations Sustainable Development Goals (SDGs) [1]. There is usually a gap between knowledge and evidence production, on one side, and its implementation, on the other. This gap hinders the spread of sustainable evidence-based practices and programs to the most in need populations [2]. For example, around 70% of neonatal deaths is caused by the reduced coverage of effective yet simple and non-capital-intensive interventions [3]. This example highlights the significant impact that narrowing this gap could have on strengthening public health systems and improving health outcomes.

Healthcare interventions proven effective should be expanded into broader policies and practices. To that end, related to implementation and translational sciences, an important concept emerges in the literature: *Scaling-up* [4, 5]. According to the World Health Organization (WHO) [6], scaling up is defined as “deliberate efforts to increase the impact of successfully tested health innovations so as to benefit more people and to foster policy and programme development on a lasting basis”. A successful expansion of local projects to national programs depends on several factors, including (but not restricted to) political aspects, logistic challenges, staff support, and acceptance by the local society [5].

To critically reflect on how to successfully scale-up of projects or adapt programs, it is necessary to identify favoring and hindering factors as analyzed in different implementation contexts across the scientific literature. The current available publications on this topic are dedicated to gather the factors specific for a setting and/or health condition. The aim of the present review is to document the type of public health programs which have been scaled up, describe the favoring and hindering factors of this process, and critically analyze the findings about it. A scoping review was conducted to systematically map the research done about factors that promote or hinder the process of scaling-up to national level, mentioning the type of program to be scaled and the methodology used in the included articles.

## Methodology

The research method is the scoping review given the research question and objectives of this project, which uses a systematic, rigorous, and transparent methodological approach as of a systematic literature review but does not necessarily assess the quality (“risk of bias”) of the original articles [7]. The research question guiding this study is: “What is documented in the international scientific literature about favoring and hindering factors of scaling-up public healthcare programs?”. Therefore, the overall objective of the present study is to systematically review the international scientific literature on scaling up public healthcare programs to national level to map the favoring and hindering factors of this process and to learn for future attempts. The Preferred Reporting Items for Systematic reviews and Meta-Analyses extension for Scoping Reviews (PRISMA-ScR) Checklist was also followed as a reporting guideline and is provided as an additional file (Additional file 1) [8].

Published articles (including thesis and dissertations), that used qualitative, quantitative, mix-methods, or systematic literature research approaches, published in English, Portuguese, Spanish or German, and were available on the internet were eligible through the search process.

A sensitive search was conducted in the following databases in early 2023: Medline (PubMed), Web of Science (WoS), Cochrane Library, Scopus, Scielo, Virtual Health Library (VHL), and Google Scholar. No restrictions were placed by date of publication or geographic region. Searches were performed and double-checked until consistent results were obtained by two authors (RCFR and TRR). One reviewer (RCFR) screened title, abstracts and full text. The preselected studies were then screened by a second reviewer (TRR). In cases where uncertainty arose, any disagreements were addressed through further discussion and resolution involving both authors. If consensus could not be reached, a third reviewer (AK) was consulted.

To identify the search terms, the population-concept-context (PCC) framework was applied as a strategy of literature research [8]. The key points are presented in Table 1. Free terms were used in all databases and combined With MeSH and DECs terms when applicable according to the database. The Google Scholar search was conducted by the reading of the first 200 titles, where no more additional relevant publications were captured (saturation point). The final search strategy for all considered databases is provided in additional file (Additional file 2).

**Table 1:** Framework, key-terms, and categories based on the research question

Framework, Key-terms, Search category	Free terms	MeSH terms	DECs terms
Population, Healthcare program, Health related programs	Government program National health program Health policy Policy making Program development Health service research Healthcare program Pilot program Research program Public healthcare program National health service Public healthcare project National health policy Policy and program development Community healthcare program Community health service	Government Programs National Health Programs Health policy Policy Making Program Development Health services research	Health programs and plans Government program National health program Health policy Policy making Programme development Health services research
Concept, Scaling up, From research to practice	Translational research Implementation science Scaling up Expand Implementation strategy	Translational Research, Biomedical, Implementation science	Implementation science Translational research
Context, Barriers and facilitators, Associated factors (barriers and facilitators)	Difficult Hindering factor Negative factor Limitation Barrier Challenge Problem Favoring factor Facilitator Enabler Success Barrier and facilitator	Not identified	Not identified

All the screening, selection and data extraction steps were conducted in Microsoft Excel (version 2023). Excel was also used to identify duplicates and Endnote as the reference management software.

The data extraction form of the Joanna Briggs Institute’s manual [7] served as the primary tool for data extraction and was adapted to reach the research objectives. An initial draft was piloted by one author (RCFR) and then revised by a second author (TRR). A total of five studies were piloted and reviewed, with additional information added as necessary. The extracted variables have been systematically categorized into four distinct groups: (i) General article information, encompassing essential details such as authorship, publication year, title, geographical location of the scaling-up endeavor, and the affiliations of the authors, along with the articulated objectives and terminological nuances; (ii) Methodological particulars, delineating the study design, sample size, research strategy, and the criteria employed for inclusion and exclusion, alongside an assessment of the methodological quality; (iii) Characteristics pertaining to the program earmarked for expansion, delineating the target demographic, the program’s background, the nature of the scaling-up initiative, the modalities involved in the scaling-up process, and the temporal trajectory of the complete scaling-up endeavor; (iv) A comprehensive examination of both impeding and facilitating factors, encompassing a spectrum of categories as established by the ExpandNet framework for scaling up health interventions. The final version of the blank data extraction form can be consulted in Additional file 3. Due to the nature of the scoping review methodology, no formal assessment of the study quality was conducted. However, the quality of the included articles was evaluated based on their respective research designs, and any identified limitations were documented.

**Fig. 1:**
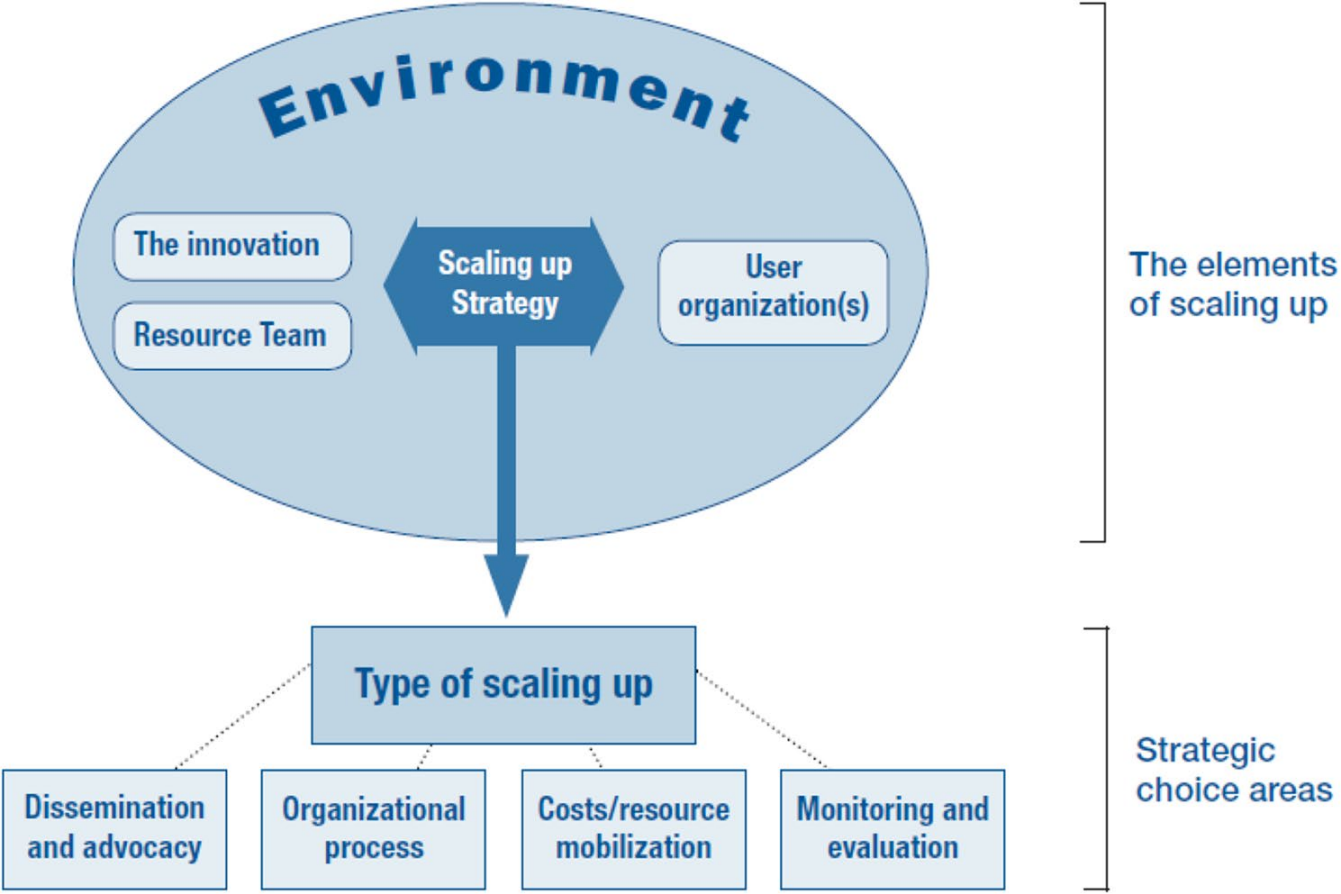
ExpandNet/WHO framework for scaling up

The favoring and hindering factors were identified using the WHO-ExpandNet framework [6]. Developed in 2010 by WHO and partners, this framework promotes a systematic approach to scaling-up by dividing the process into elements and strategic choice areas (see Fig. 1). It highlights five key elements (the innovation, user organization, environment, resource team, and type of scaling-up) and has been widely applied to support implementation and policy planning in global health.

## Results

### Identification and selection of studies

Applying the final syntax in all databases retrieved 7,027 titles. There were 1,305 duplicates, making 5,722 documents available for further assessment.

**Fig. 2:**
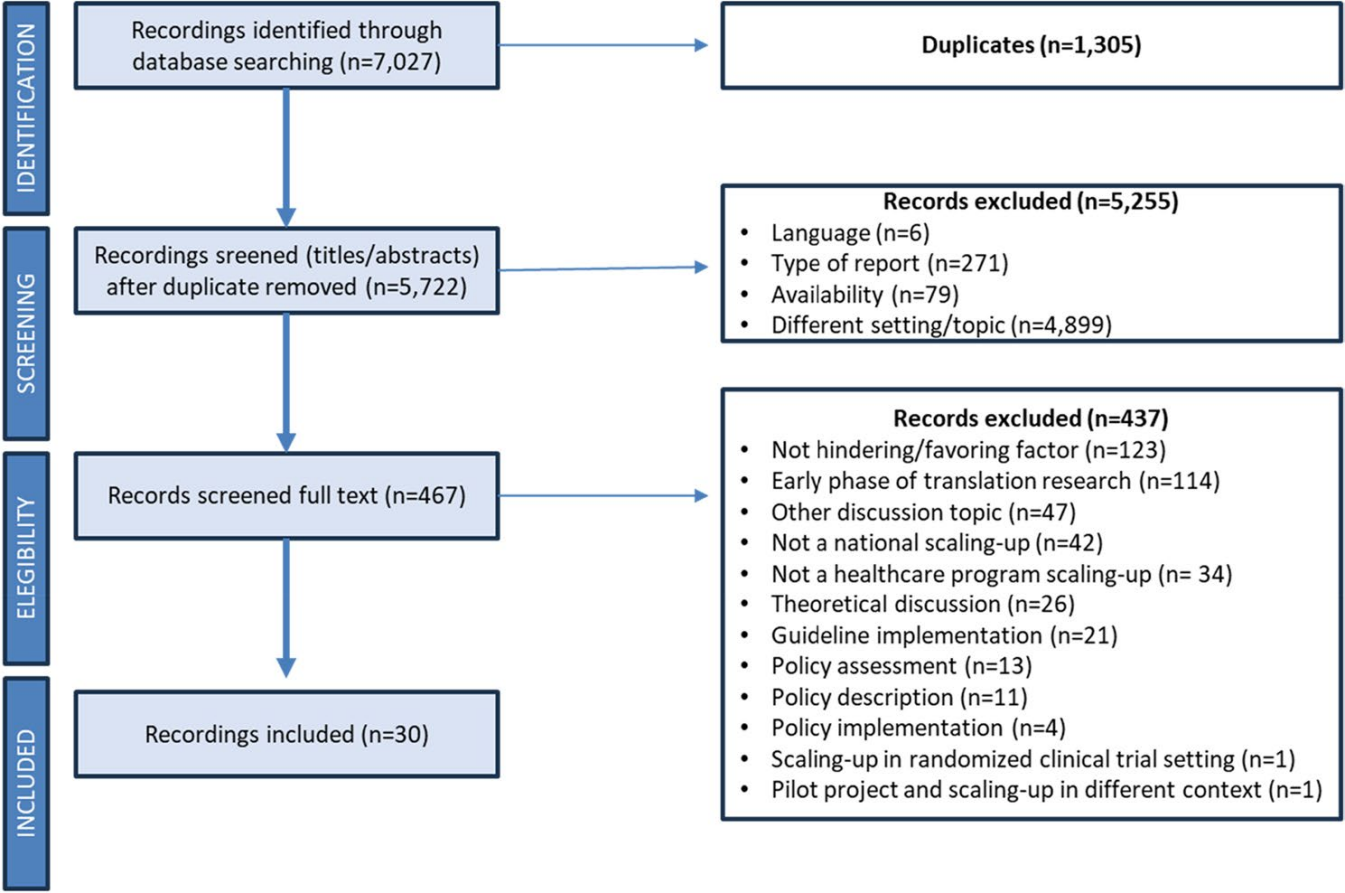
PRISMA flow diagram

In the screening phase by title and abstract, documents that did not meet the following inclusion criteria were excluded: (i) type of document (*n* = 271), (ii) different objective, based on title and abstract (*n* = 4,899), (iii) absence of available abstract and/or complete document (*n* = 79), and (iv) different language (*n* = 6). After the initial screening phase, all the remaining 467 articles were fully read and assessed considering the connection With the research question. At this stage, 437 were excluded, as they did not correspond to the research question. Based on this process, 30 articles were included in this review. Figure 2 shows the flow using the unified PRISMA flowchart [8].

### Characteristics of included studies

The included articles on scaling-up public health programs utilized diverse research methodologies, with qualitative research being the most common, often employing in-depth interviews or mixed qualitative strategies. Five articles conducted systematic literature reviews with clearly defined inclusion and exclusion criteria but lacked quality evaluation criteria, categorizing them as scoping reviews. Other study types included descriptive studies, mixed methods, secondary data analysis, and one non-systematic literature review. The articles covered various countries, with eleven featuring a multi-country perspective mostly from Low- and Middle-Income Countries (LMICs), such as Nigeria, Uganda, India, and Brazil, and many authors affiliated with U.S. institutions. Nineteen articles focused on single-country studies, predominantly in African nations, with a mix of local and international authorship. The authors frequently examined the terminology and duration of the scaling-up process, using terms such as “expansion,” “implementation,” “roll-out,” and “diffusion,” and specifying the process duration in seven articles, which ranged from short- to mid-term (under 10 years) to long-term (over 10 years). Additionally, a few articles explicitly mentioned horizontal and vertical scaling-up, which are key concepts within the WHO-ExpandNet framework. These dimensions refer, respectively, to the replication or expansion of innovations across sites (horizontal) and to their institutional integration through policy, budgetary, or systemic changes (vertical).

Table 2 shows the programs scaled up in the included articles. A reference list with the IDs of the included articles and the definitions used for classification of the programs are included as additional files (Additional files 4 and 5, respectively).

**Table 2:** Public health care programs that have been scaled-up

ID	Country	Public healthcare program	Target population	Terminology	Definition	Type of scaling-up	Steps to the National level
	(Year of publication)						
1	Estonia (2015)	Sexual and reproductive health	Adolescents	Scaling-up	1	Vertical, Horizontal	►Pilot project
							►Start-up activities
							► Replicating to new geographic areas, until reach national level
							►Institutionalization through (1) Estonian Sexual Health Association (ESHA) and personnel’s’ lobby for political commitment and funding, (2) legislation change to establish Estonian Health Insurance Fund (EHIF), and (3) inclusion of the young clinics (YCs) in the reimbursement system of EHIF
							►National scale-up
2	Kenya (2011)	Sexual and reproductive health	HIV-negative men	Scaling-up/Translating Research	2	Vertical, horizontal and depth	►Government leadership: The Kenyan Ministry of Health and the National AIDS and sexually transmitted infections (STIs) Control Programme (NASCOP) began providing leadership on medical for HIV prevention before the conclusion of the randomized controlled trials mentioned above and before WHO issued its recommendations in 2007
							►After successful pilot project, Kenya’s Director of Medical Services issued a statement calling for the establishment of a national male circumcision (MC) task force to advise the government on how to proceed.
							►Development of national policy (‘‘National Guidance for Voluntary Male Circumcision in Kenya’’)
							►National scale-up
3	China (2010)	Sexual and reproductive health	Female sex workers, Men who have sex with men (MSM)	Scaling-up	2	Vertical, Horizontal	►Surveys conducted in the 1990 s indicated low rates of condom use and very high rates of sexually transmitted diseases (STDs)
							►Monitoring HIV epidemic among female sex workers in 1995 at 13 sentinel surveillance sites
							►China Center for Disease Control and Prevention (CDC) conducted a behavioral intervention study to promote condom, use and increase HIV/STD awareness.
							►World Bank financed the pilot project
							►Adjustments to reach a self-sustainable project
							►Scale-up in some states
							►National scaling-up, with chances in policy
4	Nigeria (2017)	Sexual and reproductive health	Pregnant women - rural context	Scaling-up	2	Horizontal	►Pilot project
							►National scale-up
5	South Africa (2020)	Sexual and reproductive health	Women	Scaling-up, Uptake	2	Horizontal, Depth	Not mentioned
6	Zambia (2015)	Sexual and reproductive health	Neonatal	National roll-out	2	Vertical, Horizontal, Depth	►Pilot project
							►National policy
							►National scale-up
7	Mozambique (2015)	Sexual and reproductive health	Adolescents	Scaling-up	1	Vertical, Horizontal	►Intersectoral Committee for the Development of Youth and Adolescents was established involving the ministries of health, education, youth, women’s affairs, labor, and environmental action as well as non-government organizations (NGOs) and faith-based organizations National.
							►Plan for the Development of Adolescents and Youth. Programa Geração Biz (PGB)
							►Pilot PGB activities
							►Small scale pilot expansion
							►World Bank provided fund to integrate HIV test and care
							►National scale-up
8	Canada (2018)	Digital tools	General population	Expansion	1	Vertical, Horizontal	►Pilot project
							►Explore potential barriers and facilitators factors to national scale-up: e.g., Think Tank
							►National scale-up
9	India (2021)	Digital tools	Front-line health workers	Scaling-up	2	Vertical, Horizontal, Depth	►National Family Health Survey reported that utilization of key maternal and newborn health services is variable and characterized by breaks in the continuity of care
							►Pilot projects (different projects)
							►National scale-up
10	South Africa (2021)	Digital tools	Pregnant women, Children	Scaling-up	2	Horizontal	Not mentioned
11	Multi-country (2012)	Breastfeeding	Lactating women	Scaling-up	?	Vertical, Horizontal	Not mentioned
12	Multi-country (2008)	Breastfeeding	Pregnant and lactating women	Scaling-up	2	Vertical, Depth	Not mentioned
13	Malawi (2019)	Breastfeeding	Neonatal	Scaling-up	1	Horizontal	►International policy (WHO/UNICEF)
							►Largely implemented in several countries
							►Systematic Literature Review
							►International guideline on how to implement such program (WHO/UNICEF)
							►Revitalization of an old local policy
							►National scale-up
14	Multi-country (2013)	Community Health Workers	General population in LMIC	Scaling-up/Diffusion/Dissemination	2	Vertical, Horizontal	Not mentioned
15	Zambia (2017)	Community Health Workers	Population with reduced access to health services	Scaling-up	2	Horizontal, Depth	►National Community Health Worker Strategy (NCHWS)
							►Under the NCHWS, the Ministry of Health is using a phased approach to recruit, train, and deploy a workforce of 5000 Community Health Workers
							►Pilot project to check community acceptability
							►National scale-up
16	Multi-country (2015)	Community Health Workers	Cardiovascular disease risk population in LMIC	Scaling-up	2	Horizontal	Not mentioned
17	Multi-country (2013)	Preventive of Chronic disease	General high-income population	Scaling-up	2	Vertical, Horizontal	Not mentioned
18	India (2018)	Community-based programme	Neonatal and children	Scaling-up	2	Horizontal, Depth	►Different national policies (i.e., National Policy on Children)
							►Pilot project
							►Consolidation of the design and strengthening institutional capacity
							►Universalization
							►Restructuring and mission mode
							►Monitoring and evaluation
							►National scale-up
19	Rwanda (2014)	Family planning	Women in reproductive age	Scaling-up	2	Vertical, horizontal, depth	►Pilot project
							►To integrate Standard Days Method (SDM) into the new family planning policies, norms, training curricula, and management information and logistics systems
							►National scale-up
20	Multi-country (2021)	Family planning	General population	Scaling-up	2	Horizontal	Not mentioned
21	Iran (2021)	Family program	Families	Implementation	2	Horizontal	Not mentioned
22	Peru (2005)	Integrated Childhood Illness	Children under 5 years old	Scaling-up	2	Vertical, Horizontal	►International guideline
							►Pilot project
							►National scale-up
23	Ethiopia (2017)	Mental health	General population	Scaling-up	2	Vertical, horizontal, depth	►Pilot project (WHO collaboration - commitment to scale up)
							►Research-led programs to implement and evaluate integrated mental health care are also underway in Ethiopia, including the Programme for Improving Mental health care (PRIME), the Africa Focus on Intervention Research for Mental Health (AFFIRM) and the ‘Emerging mental health systems in LMICs’ (Emerald) programme.
							►National scale-up
24	Multi-country (2012)	Nutrition	Pregnant women	Scaling-up	2	Horizontal, Depth	►National program
							►Survey to assess the impact of the absence of such intervention
							►Include the proposed intervention into a broader program
							►Pilot project
							►National scale-up
25	Multi-country (2015)	Not specific	General population	Scaling-up	2	Vertical, Horizontal	Not mentioned
26	Australia (2020)	Not specific	Not specific	Scaling-up	2	Vertical, Horizontal	Not mentioned
27	Multi-country (2012)	Not specific	General population in LMIC	Scaling-up	2	Vertical, Horizontal	Not mentioned
28	Multi-country (2010)	Not specific	Not specific	Scaling-up	2	Vertical, Horizontal	Not mentioned
29	Australia (2021)	Not specific	Not specific	Scaling-up	2	Vertical, Horizontal	Not mentioned
30	Multi-country (2019)	Not specific	Not specific	Scaling-up	2	Vertical, Horizontal	►Ghana: The process included piloting, field demonstrations, resource assessment, leadership development, building and equipping facilities and assigning health staff, training counterparts and deploying volunteers. Authors credit its success to consensus building, a sense of ownership among the targeted communities, the presence of change agents, credibility of the change and demonstration of feasibility in the communities. They also credit the involvement of all levels of bureaucracy in the change.
							►Canada: Scale-up involved establishing action zones, a central support team, school facilitators and stakeholder teams, a planning guide and resource directory, and bins filled with exercise equipment. This program credits its success at the micro-level to training and resourcing teachers, supportive school policies and sustained implementation. At the macro-level, they credit their success to multisectoral partnerships and embedded knowledge exchange mechanisms.

1 = scale-up approaches have been defined by the authors

2 = scale-up approaches classified based on established definition in the present study

Among the included articles, four publications were published before the framework was produced [9–12], and 21 documents did not use the WHO-recommended categories [2, 13–32]. In both cases, we categorized the factors according to the framework criteria. For the purpose of this scoping review, favoring and hindering were defined as follows [6]:

- Favoring factors: Any factor that enables the implementation of evidence-based interventions at scale.
- Hindering factors: Any factor that obstructs the implementation of evidence-based interventions at scale.

**Fig. 3:**
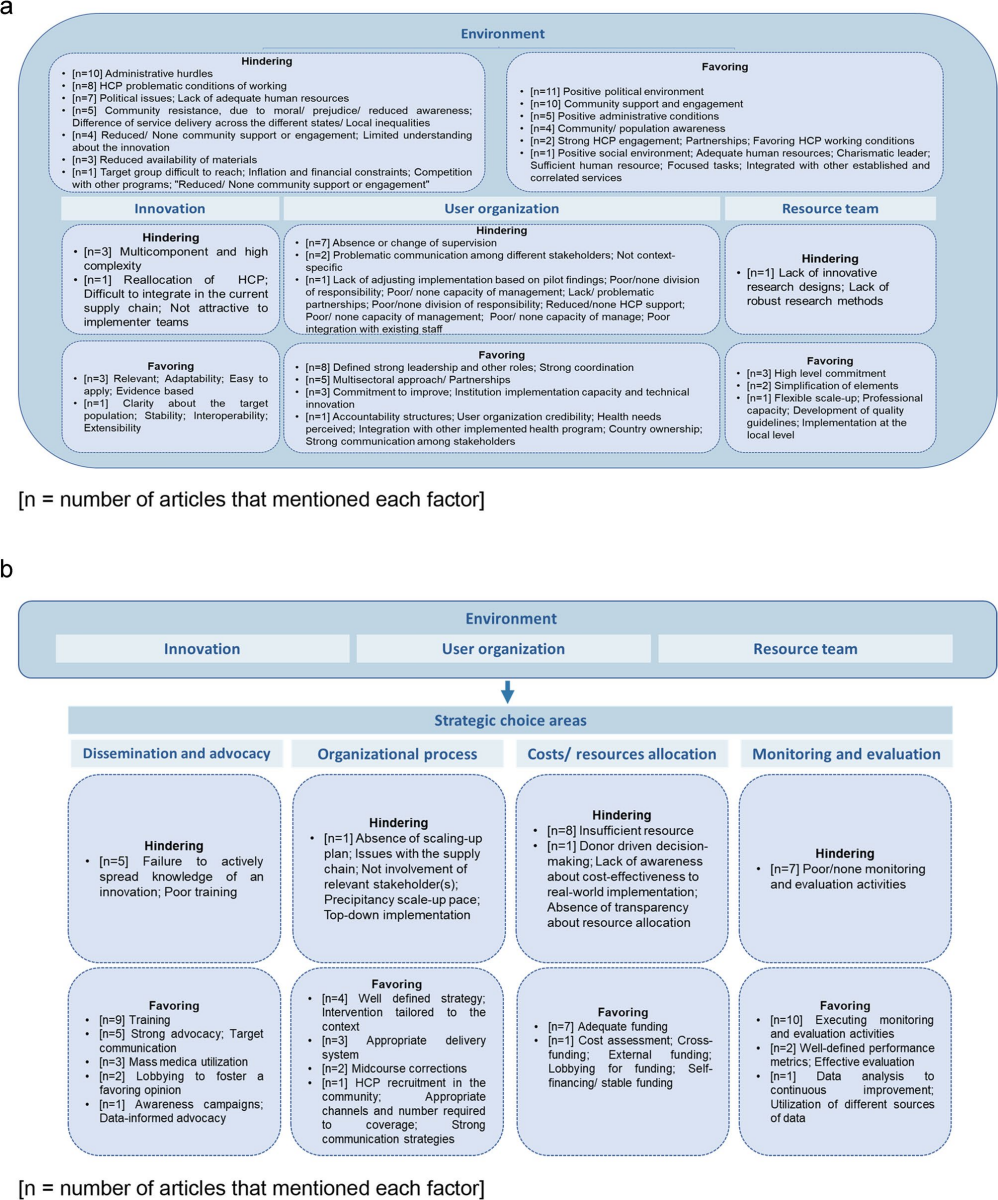
**a**. Hindering and favoring factors of the scaling-up process according to the “elements of scaling-up” from ExpandNet/WHO framework. **b**. Hindering and favoring factors of the scaling-up process according to the “strategic choice areas” from ExpandNet/WHO framework

Figure 3a and 3b summarize all findings in this framework. Additional file 6 describes all favoring and hindering factors.

### Elements of scaling-up

“*Environment*” refers to the implementing organization’s external conditions which may positively or negatively affect the prospects for scaling up [6]. Seventeen articles mentioned at least one environmental factor as a barrier [10, 12, 15–17, 19, 20, 23–27, 29, 32–35]. Administrative hurdles were the most relevant factor, mentioned by 10 articles, including insufficient infrastructure. As stated by Okeke et al. (2017) a poor clinical infrastructure hampered midwives from delivering appropriate care in Nigeria. A positive political environment was a favoring environmental feature in several other countries (*n* = 11) [4, 9, 16, 24, 25, 27, 30, 31, 34, 36, 37]. Political will was mentioned as a major enabling factor by Chandra-Mouli et al. [36], who attributed part of the scaling-up success in Mozambique to the political momentum, which engaged local institutions and identified and addressed local contextual factors to facilitate the programme.

“*Innovation*” is defined as the new intervention or group of new interventions that will be scaled, for example maternal programs and Community Health Workers (CHW) in primary healthcare [6]. The multicomponent and high-complexity features of innovations can be barriers related to the innovation itself [2, 27, 36]. For example, Chandra-Mouli et al. [36] mentioned that the expansion of an adolescent sexual and reproductive health program (Programa Geração Biz) demanded an intersectoral approach, including ministries of health, education, youth, women’s affairs, labor, and environmental action, as well as the participation of NGOs and faith-based organizations. This specific feature of the intervention increased the challenges to the scale-up. Regarding the favoring factors of the process, some articles highlighted particular features as being relevant [29, 36, 37], such as: adaptable [29, 34, 36], easy to apply [30, 36, 37], and evidence based [29, 34, 37]. For instance, Chandra-Mouli et al. [36] mentioned that in Mozambique prior to the inception of the Programa Geração Biz, a national needs assessment was conducted to identify the adolescents’ needs regarding sexual and reproductive health.

“*User organization(s)*” are the institutions or organizations that accept and implement the innovation on a large scale [6]. Some articles (*n* = 12) mentioned at least one feature of the user organizations that might affect the expansion of the innovation [2, 10, 16, 20, 23–28, 32, 33]. Absence or change of supervision was often mentioned (*n* = 7) [2, 10, 16, 24, 26, 28, 33]. For instance, the scale-up of a mental healthcare program in Ethiopia was threatened by the reduced availability of adequate supervisors for the newly trained primary care workers (Hanlon et al. 2017). In contrast, 16 articles highlighted how the user organization can be positive for innovation expansion, particularly strong leadership and other roles (*n* = 8) [9, 11, 18, 21, 22, 30, 31, 37]. Also, strong coordination (*n* = 8) [9, 11, 14, 22, 24, 25, 31, 36] was mentioned as favoring factor. In this sense, the success of the African National Female Condom Programme in South Africa was partially due to the coordinated partnership between the National Department of Health and NGOs that could reach remote areas and properly approach the target group (sex workers, men who have sex with men, and youth).

“*Resource team*” refers to all individuals and organizations that are connected to the scaling-up process, being formally or informally in this position [6]. Only one article mentioned this category as a barrier [2] with the argument that the lack of robust research methods and innovative research designs can increase the challenges to scaling up an innovation to the national level. Five articles mentioned factors related to the resource team that facilitate the expansion of innovations [21, 31, 33, 35, 37], such as the high-level commitment of the team. Milat et al. (2015) mentioned the active engagement of a range of implementers to be an important feature for the success of the scale-up process. Other factors included simplification of elements [33, 37] and high professional capacity [31].

### Strategic choice areas

“*Dissemination and advocacy*” is defined as any action conducted that may increase the support and reduce opposition among individuals, groups, or institutions. It can be personal, such as training, technical assistance, or impersonal, for example web sites, publications, policy briefs, and toolkits [6]. Five articles mentioned the failure to actively spread knowledge of an innovation as a hindering factor [2, 12, 14, 20, 23]. Rou et al. (2010) mentioned specifically the hampering effect of poor communication in different government areas and Beksinska et al. (2020) the lack of communication with the target communities. Fourteen publications mentioned favoring factors connected with communication and dissemination [9, 13, 14, 18, 21, 22, 24, 25, 28, 31, 34–37]. Staff training was the most prominent factor for a successful scale-up process [9, 13, 14, 22, 24, 25, 28, 34, 37]. According to Mwaikambo et al. (2021), appropriate training of administrative staff and healthcare professionals was imperative to strengthen the team’s capacities and for the innovation’s successful expansion. Strong advocacy, including communication and mass media campaigns to mobilize mothers in target areas about the relevance of breastfeeding, was also mentioned [9, 18, 21, 25, 31].

The “*organization process*” is any feature related to the scale-up activities, for example, the scope and pace of scaling up, the number of agencies involved, whether it is centralized or decentralized, whether it is an adaptive or fixed process, and whether it is participatory or donor/expert-driven [6]. Four articles mentioned features of the organization process with a potential negative effect on the scaling-up [14, 20, 29, 32]. For example, Zomahoun et al. (2019) mentioned that a topdown implementation is not a valid method to implement an innovation at the national scale. According to the authors, due to high-level decisions, population-wide interventions may not reflect the specific needs, preferences, or values of the targeted end-users, hindering the entire process. Eight publications mentioned factors that may favor the process [4, 9, 18, 24, 25, 28, 31, 33]. For instance, the innovation must be tailored to the context where it is going to be applied [21, 24, 25, 31]. Perez-Escamilla et al. (2012) stated that scaling-up processes that adapt the innovation to address cultural beliefs have a better chance of being successfully implemented.

“*Costs/resources mobilization*” is part of the scaling-up strategy focused on the assessment of the budget al.location for the scaling-up process and its sustainable implementation [6]. Ten articles mentioned poor resource allocation as a hindering factor [2, 10, 17, 20, 24, 27, 29, 30, 32, 34]. Mehrolhassani et al. (2021) [20] noted that the budget required for the implementation of a family physician programme in Iran was approved but not budgeted for due to various reasons (including a change of ministers and governments). On the other hand, the proper funding of the scaling-up project acts as a favoring factor [9, 11, 18, 25, 30, 31, 36]. As discussed by Chandra-Mouli et al. [36], the resources came from a variety of international funding bodies as well as from the government of Mozambique.

“*Monitoring and evaluation*” are activities focused on the tracking of the progress and success of the scale-up project [6]. Poor monitoring and evaluation activities were associated with barriers to the successful scale-up of interventions [12, 14, 16, 26, 27, 30, 31]. For Rao and Kaul (2018), the lack of attention to monitoring public awareness of early childhood development has contributed in India to less public demand for an intervention related to this issue thus hindering the scale-up process. In contrast, projects that considered monitoring and evaluation activities to be important found this to be a favoring factor for large scale implementation [9, 21, 22, 24, 25, 28, 31, 33, 36, 37].

## Discussion

This scoping review provides valuable information about the factors that influence the scaling-up processes of public health programs from pilot or project level to national programs. The WHO-ExpandNet framework is a useful tool for categorizing enabling and limiting factors shaping the scale-up process.

The results indicate that the most frequently mentioned categories of *hindering factors* for scaling-up processes were poor “user organization”, “institutional environment”, and “resource mobilization.” Regarding the user organization category, the most common problem was the absence or change of staff supervisors. In the category environment, the administrative hurdles, such as bureaucracy or insufficient infrastructural development, were frequently mentioned. In the category of resource mobilization particularly insufficient resources and a lack of budgeting were highlighted as hindering factors.

*Favoring factors* were particularly identified in the categories of “user organization”, “environment”, and “dissemination and advocacy”. Regarding a user organization’s strong leadership, well-defined roles as well as good coordination were factors facilitating a scale up to the national level. In the category of institutional environment, a positive political environment and community support and engagement were often mentioned as enabling factors. Also, strong advocacy and program dissemination through staff training and mass media involvement were relevant features of a successful uptake; however, hindering and favoring factors do not stand alone but are correlated; they interact, enhance, or hinder each other. While this review focused on identifying individual favoring and hindering factors, it is important to recognize that these elements often interact in complex and context-dependent ways. For example, strong leadership may be more effective when coupled with sustained political will and adequate resource mobilization. Future research could further explore how these dynamics shape scaling-up trajectories, helping to identify combinations of factors that are particularly critical for success or failure.

An example is given by a study on the scaling up of a dengue prevention project in Brazil with impregnated window screens (not included in this review as it did not reach the national program level) [5]. The long bidding process to identify local enterprises to produce tailor-made window screens at a reasonable price, the bureaucratic challenges, and the inflexibility of the national program to provide incentives for vector control staff were prohibitive for implementing this highly efficacious measure in the national vector control program.

Regarding resource allocation, it is expected that the organizing team of the scaling-up process estimates the necessary budget for an expansion project before starting. One example of this practice is presented in the translational process of the visceral leishmaniasis elimination initiative on the Indian Subcontinent [38]. According to the authors, the close coordination and partnership among international institutions (e.g., the WHO) and local stakeholders, particularly national program managers, were imperative features of the success of the translation of research projects into policy and practice. The participating institutions, (e.g., the Ministries of Health, the National Institutes of Health, universities, and international organizations) were aware of the new strategy’s cost implications and were able to take up the challenge. In other cases, even when the research team calculates the expected costs for the program, the allocation may not happen (see Mehrolhassani et al. (2021)) [20]. It is also important to note that the scaling-up process normally takes time, and some factors identified at the beginning may change, e.g., due to a change of health authorities or factors such as increased inflation, war, natural disasters, disease outbreaks, and others.

Regarding the programs to be scaled-up, it is interesting to note that gender issues and reproductive health are the most common projects described in the publications. It seems justified that these topics have to be discussed particularly – but not only – in LMICs, where the incidence of early pregnancies, sexually transmitted infections, and maternal mortality is high [39–42].

The findings of this scoping review do not suggest changes to the WHO-ExpandNet framework but rather reinforce its utility for organizing and interpreting real-world evidence on scale-up processes. Public health practitioners and policymakers may benefit from using the framework as a practical tool to identify and plan for key enabling and hindering factors in the design and implementation of scale-up strategies. In particular, the evidence summarized here may assist in diagnosing potential bottlenecks related to user organization capacity, institutional environments, and funding mechanisms. The recurrent emphasis on leadership, coordination, and contextual adaptability further offers guidance for implementation planning in diverse LMIC settings. Nonetheless, the use of these findings should consider local specificities, and further research is needed to capture lessons from unsuccessful scale-up attempts.

### Limitations of the study

Although the scoping review methodology does not require a quality assessment of the included articles, general study design limitations identified were noted. It is valid to highlight that the absence of the bias assessment may represent a relevant inconsistency with the good practices for Systematic Literature Reviews, according to Preferred Reporting Items for Systematic Reviews and Meta-Analyses (PRISMA). However, some of the seven Systematic Literature Reviews (which are supposed to include a bias assessment) did not even mention it. On the other hand, for operational research, the assessment of bias is much less relevant than for clinical trials or randomized community trials. This is particularly true for qualitative research with in-depth interviews and Focus Group Discussions (FGDs) including a limited number of selected interview partners. These studies are not representative, and the level of bias will be assessed by the participating group members, by the research team, and by comparing with quantitative studies (triangulation).

This research focused on the collection of information about favoring and hindering factors of scaling-up pilot projects or international programs to a national level. Therefore, projects on a horizontal scale-up (i.e., the replication of a pilot project in another region or country) or on a middle-level vertical scale up (where the full scaling-up process towards the national level were not completed) were not included in this scoping review but would warrant another study. Also, the documentation of unsuccessful scaling-up attempts would be important to learn from.

## Conclusions

Different factors may affect the scaling-up of innovations to national program levels in different settings. The WHO-ExpandNet framework is a useful tool to foresee potential barriers and favoring factors. It is important to consider that the expansion of an intervention is not a rigid process, and one determining factor may affect others. Furthermore, scaling-up is a process that may take considerable time, so that the mapping of factors at the beginning is just a snapshot of one specific moment. However, the analysis of possible favoring and hindering factors is useful to be prepared for the complexity of the process.

The results of this scoping review are of interest to different stakeholders, including decision-makers, researchers, and communities. Avenues for future research include the investigation of an association between different factors, the assessment of the scaling-up process in levels besides the national level, and the review of experiences which failed.

## Supplementary Information

Additional file 1: PRISMA-ScR checklist

Additional file 2: Search strategy

Additional file 3: Data extraction form

Additional file 4: IDs of the included articles

Additional file 5: Glossary of terms

Additional file 6: List of enabling and hindering factors

## Data Availability

The datasets used and/or analyzed during the current study are available from the corresponding author on reasonable request.

## Abbreviations

*CHW*: Community Health Workers
*LMIC*: Low- and middle-income countries
*NGOs*: Non-Governmental Organizations
*SDGs*: Sustainable Development Goals
