# Supplementary material for: From intervention studies to national programs, what are the favoring and hindering factors? a scoping review
Source: BMC Public Health. 2025 Oct 28;25:3623. doi: 10.1186/s12889-025-24770-1 (PMC12560514; doi:10.1186/s12889-025-24770-1)
Supplement: Supplementary file 2 — Additional file 2: Search strategy [file 12889_2025_24770_MOESM2_ESM.docx]

**Additional file 2. Search strategy.**

| **Database** | **Final syntaxis** | **Number of articles** |
| --- | --- | --- |
| PubMed | ("government programs"[Mesh] OR "national health programs"[Mesh] OR “health policy”[MeSH Terms] OR "policy making"[Mesh] OR "program development"[Mesh] OR “health services research”[MeSH Terms] OR “healthcare program*”[tw] OR “pilot program*”[tw] OR “research program*”[tw] OR “public healthcare program*”[tw] OR “national health service*”[tw] OR “public healthcare project*”[tw] OR “national health polic*”[tw] OR "polic* and program* development"[tw] OR “community healthcare program*”[tw] OR “community health service*”[tw]) AND ("translational research, biomedical"[Mesh] OR “implementation science”[MeSH Terms] OR “scaling up”[tw] OR “expand”[tw] OR “implementation strateg*”[tw]) AND (difficult*[tw] OR “hindering factor*”[tw] OR “negative factor*”[tw] OR limitation*[tw] OR barrier*[tw] OR challenge*[tw] OR problem*[tw] “favoring factor*”[tw] OR facilitator*[tw] OR enabler*[tw] OR success[tw] OR “barrier* and facilitator*”[tw]) | **644** |
| Web of Science | (“government program*” OR “national health program*” OR “health polic*” OR “policy making” OR “program* development” OR “health service* research” OR “healthcare program*” OR “pilot program*” OR “research program*” OR “public healthcare program*” OR “national health service*” OR “public healthcare project*” OR “national health polic*” OR “polic* and program* development” OR “community healthcare program*” OR “community health service*”) AND (“translational research” OR “implementation science” OR “scaling up” OR “expand” OR “implementation strateg*”) AND (difficult* OR “hindering factor*” OR “negative factor*” OR limitation* OR barrier* OR challenge* OR problem* “favoring factor*” OR facilitator* OR enabler* OR success OR “barrier* and facilitator*”) | **1,033** |
| Cochrane Library | (“government program*” OR “national health program*” OR “health policy” OR “policy making” OR “program* development” OR “health service research” OR “healthcare program*” OR “pilot program*” OR “research program*” OR “public healthcare program*” OR “national health service” OR “public healthcare project” OR “national health policy” OR “policy and program* development” OR “community healthcare program*” OR “community health service”) AND (“translational research” OR “implementation science” OR “scaling up” OR “expand” OR “implementation strategy”) AND (difficulty* OR “hindering factor” OR “negative factor” OR limitation OR barrier OR challenge OR problem OR “favoring factor” OR facilitator OR enabler OR success OR “barrier and facilitator”) | **335**  (6 Cochrane review + 329 trials) |
| Scopus | ("government program*" OR "national health program*" OR "health polic*" OR "policy making" OR "program* development" OR "health service* research" OR "healthcare program*" OR "pilot program*" OR "research program*" OR "public healthcare program*" OR "national health service*" OR "public healthcare project*" OR "national health polic*" OR "polic* and program* development" OR "community healthcare program*" OR "community health service*") AND ("translational research" OR "implementation science" OR "scaling up" OR "expand" OR "implementation strateg*") AND (difficult* OR "hindering factor*" OR "negative factor*" OR limitation* OR barrier* OR challenge* OR problem* "favoring factor*" OR facilitator* OR enabler* OR success OR "barrier* and facilitator*") | **560** |
| VHL | ("health programs and plans" OR "government program" OR "national health program" OR "pilot program*" OR "programa* piloto*" OR "research program*" OR "programa* de pesquisa" OR "programa* de investigación" OR "public healthcare program*" OR "programa* de saúde pública" OR "programa* de salud publica" OR "national healthcare service*" OR "serviço* naciona* de saúde" OR "Servicio* Nacional* de Salud" OR "public healthcare project*" OR "projeto* de saúde pública" OR "projecto* de salud publica" OR "health policy" OR "national* health polic*" OR "política* naciona* de saúde" OR "política* nacional* de salud" OR "policy making" OR "polic* and program* development" OR "desenvolvimento de política* e programa*" OR "desarrollo de política* y programa*" OR "programme development" OR "health services research") AND ("scaling up" OR "escala" OR "ampliación" OR "implementation science" OR "translational research" OR "implementation strateg*" OR "estrategia* de implementação" OR "estrateg* de implementación" OR "expand" OR "expansión" OR "expansão") AND (difficult* OR dificuldade* OR dificuldad* OR "hinder factor*" OR "fator* de impedimento" OR "factor* de impedimiento" OR "negative factor*" OR "fator* negativo*" OR "factor* negativo*" OR limitation OR limitação* OR limitación* OR "favoring factor*" OR "fator* de favorecimento" OR "factor* favorable*" OR "barriers and facilitators" OR "barreira* e facilitador*" OR "barrera* y facilitador*" OR barrier* OR barreira* OR barrera* OR facilitor* OR facilitador* OR problem* OR problema* OR success OR sucesso OR éxito OR enabler* OR challenge* OR desafio* OR desafío*) | **4,404** |
| Scielo | ("government program" OR "government programs" OR "government programme" OR "government programmes" OR "national health program" OR "national health programs" OR "national health programme" OR "national health programmes" OR "health policy" OR "health policies" OR "policy making" OR "program development" OR "programs development" OR "programme development" OR "programmes development" OR "health service research" OR "healthcare program" OR "healthcare programs" OR "healthcare programme" OR "healthcare programmes" OR "pilot program" OR "pilot programs" OR "pilot programme" OR "pilot programmes" OR "research program" OR "research programs" OR "research programme" OR "research programmes" OR "public healthcare program" OR "public healthcare programs" OR "public healthcare programme" OR "public healthcare programmes" OR "national health service" OR "national health services" OR "public healthcare project" OR "public healthcare projects" OR "national health policy" OR "national health policies" OR "policy and program development" OR "policies and programs development" OR "policy and programme development" OR "policies and programmes development" OR "community healthcare program" OR "community healthcare programs" OR "community healthcare programme" OR "community healthcare programmes" OR "community health service" OR "community health services") AND ("translational research" OR "implementation science" OR "scaling up" OR expand OR "implementation strategy" OR "implementation strategies") AND (difficulty OR difficulties OR "hindering factor" OR "hindering factors" OR "negative factor" OR "negative factors" OR limitation OR limitations OR barrier OR barriers OR challenge OR challenges OR problem OR problems OR "favoring factor" OR "favoring factors" OR facilitator OR facilitators OR enabler OR enablers OR success OR "barrier and facilitator" OR "barriers and facilitators") | **51** |
| Google Scholar | ("national health program" OR "national health programs" OR "national health programme" OR "national health programmes") AND ("translational research" OR "implementation science" OR "scaling up" OR expand OR "implementation strategy" OR "implementation strategies") AND (“barriers” OR “hindering” OR “challenge” OR “problem” OR “facilitator” OR “enabler” OR "barriers and facilitators") | **200**  (Saturation point) |
