# Supplementary material for: From intervention studies to national programs, what are the favoring and hindering factors? a scoping review
Source: BMC Public Health. 2025 Oct 28;25:3623. doi: 10.1186/s12889-025-24770-1 (PMC12560514; doi:10.1186/s12889-025-24770-1)
Supplement: Supplementary file 3 — Additional file 3: Data extraction form [file 12889_2025_24770_MOESM3_ESM.docx]

**Additional file 3. Data extraction form.**

| **General information** |
| --- |
| Study ID |
| Author (year) |
| Source |
| Country |
| Author(s) affiliation |
| Objective |
| Terminology |
| **Methodology information** |
| Study design |
| Sample size |
| Research strategy |
| Inclusion criteria |
| Exclusion criteria |
| Quality assessment |
| Other information |
| **Innovation description** |
| Program to be expanded |
| Target group |
| Type of background to the program |
| Type of scaling-up |
| Process to national scaling-up |
| Duration of the scaling-up |
| Principal feature of the program |
| **Hindering and favoring factors** |
| The innovation |
| The innovation example |
| The innovation proposed solution |
| The user organization |
| The user organization example |
| The user organization proposed solution |
| Environment |
| Environment example |
| Environment proposed solution |
| Resource team |
| Resource team example |
| Resource team proposed solution |
| Dissemination and advocacy |
| Dissemination and advocacy example |
| Dissemination and advocacy proposed solution |
| Organizational choices |
| Organizational choices example |
| Organizational choices proposed solution |
| Costs and resources mobilization |
| Costs and resources mobilization example |
| Costs and resources mobilization proposed solution |
| Monitoring and evaluation |
| Monitoring and evaluation example |
| Monitoring and evaluation proposed solution |
| Other factors |
| Other factors example |
| Other factors proposed solution |
| **Final findings** |
| How barriers and facilitators were assessed |
| Author(s)’ recommendation |
| Results |
| Discussion |
| Conclusion |
| Key message |
| Success of scaling-up |
