# Supplementary material for: From intervention studies to national programs, what are the favoring and hindering factors? a scoping review
Source: BMC Public Health. 2025 Oct 28;25:3623. doi: 10.1186/s12889-025-24770-1 (PMC12560514; doi:10.1186/s12889-025-24770-1)
Supplement: Supplementary file 4 — Additional file 4: IDs of the included articles [file 12889_2025_24770_MOESM4_ESM.docx]

**Additional file 4. IDs of the included articles.**

| **ID** | **References – Included articles** |
| --- | --- |
| 1 | Kempers J, Ketting E, Chandra-Mouli V, et al. (2015) The success factors of scaling-up Estonian sexual and reproductive health youth clinic network--from a grassroots initiative to a national programme 1991-2013. Reprod Health 12: 2. |
| 2 | Mwandi Z, Murphy A, Reed J, et al. (2011) Voluntary medical male circumcision: translating research into the rapid expansion of services in Kenya, 2008-2011. PLoS Med 8: e1001130. |
| 3 | Rou K, Sullivan SG, Liu P, et al. (2010) Scaling up prevention programmes to reduce the sexual transmission of HIV in China. Int J Epidemiol 39 Suppl 2: ii38-46. |
| 4 | Okeke EN, Pitchforth E, Exley J, et al. (2017) Going to scale: design and implementation challenges of a program to increase access to skilled birth attendants in Nigeria. BMC Health Serv Res 17: 356. |
| 5 | Beksinska M, Nkosi P, Mabude Z, et al. (2020) Lessons from the evaluation of the South African National Female Condom Programme. PLoS One 15: e0236984. |
| 6 | Shelley KD, Ansbro EM, Ncube AT, et al. (2015) Scaling Down to Scale Up: A Health Economic Analysis of Integrating Point-of-Care Syphilis Testing into Antenatal Care in Zambia during Pilot and National Rollout Implementation. PLoS One 10: e0125675. |
| 7 | Chandra-Mouli V, Gibbs S, Badiani R, et al. (2015) Programa Geracao Biz, Mozambique: how did this adolescent health initiative grow from a pilot to a national programme, and what did it achieve? Reprod Health 12: 12. |
| 8 | Liddy C, Moroz I, Joschko J, et al. (2018) Using an Integrated Knowledge Translation (IKT) Approach to Enable Policy Change for Electronic Consultations in Canada. Healthc Policy 14: 19-29. |
| 9 | Singh NS, Scott K, George A, et al. (2021) A tale of 'politics and stars aligning': analysing the sustainability of scaled up digital tools for front-line health workers in India. BMJ Glob Health 6. |
| 10 | Swartz A, LeFevre AE, Perera S, et al. (2021) Multiple pathways to scaling up and sustainability: an exploration of digital health solutions in South Africa. Global Health 17: 77. |
| 11 | Perez-Escamilla R, Curry L, Minhas D, et al. (2012) Scaling up of breastfeeding promotion programs in low- and middle-income countries: the "breastfeeding gear" model. Adv Nutr 3: 790-800. |
| 12 | Bhandari N, Kabir AK and Salam MA. (2008) Mainstreaming nutrition into maternal and child health programmes: scaling up of exclusive breastfeeding. Matern Child Nutr 4 Suppl 1: 5-23. |
| 13 | Kavle JA, Welch PR, Bwanali F, et al. (2019) The revitalization and scale-up of the Baby-Friendly Hospital Initiative in Malawi. Matern Child Nutr 15 Suppl 1: e12724. |
| 14 | Pallas SW, Minhas D, Perez-Escamilla R, et al. (2013) Community health workers in low- and middle-income countries: what do we know about scaling up and sustainability? Am J Public Health 103: e74-82. |
| 15 | Phiri SC, Prust ML, Chibawe CP, et al. (2017) An exploration of facilitators and challenges in the scale-up of a national, public sector community health worker cadre in Zambia: a qualitative study. Hum Resour Health 15: 40. |
| 16 | Abrahams-Gessel S, Denman CA, Montano CM, et al. (2015) Training and supervision of community health workers conducting population-based, noninvasive screening for CVD in LMIC: implications for scaling up. Glob Heart 10: 39-44. |
| 17 | Gaziano TA and Pagidipati N. (2013) Scaling up chronic disease prevention interventions in lower- and middle-income countries. Annu Rev Public Health 34: 317-335. |
| 18 | Rao N and Kaul V. (2018) India's integrated child development services scheme: challenges for scaling up. Child Care Health Dev 44: 31-40. |
| 19 | Igras S, Sinai I, Mukabatsinda M, et al. (2014) Systems approach to monitoring and evaluation guides scale up of the Standard Days Method of family planning in Rwanda. Glob Health Sci Pract 2: 234-244. |
| 20 | Mwaikambo L, Brittingham S, Ohkubo S, et al. (2021) Key factors to facilitate locally driven family planning programming: a qualitative analysis of urban stakeholder perspectives in Africa and Asia. Global Health 17: 75. |
| 21 | Mehrolhassani MH, Jahromi VK, Dehnavieh R, et al. (2021) Underlying factors and challenges of implementing the urban family physician program in Iran. BMC Health Serv Res 21: 1336. |
| 22 | Huicho L, Davila M, Campos M, et al. (2005) Scaling up integrated management of childhood illness to the national level: achievements and challenges in Peru. Health Policy Plan 20: 14-24. |
| 23 | Hanlon C, Eshetu T, Alemayehu D, et al. (2017) Health system governance to support scale up of mental health care in Ethiopia: a qualitative study. Int J Ment Health Syst 11: 38. |
| 24 | Victora CG, Barros FC, Assuncao MC, et al. (2012) Scaling up maternal nutrition programs to improve birth outcomes: a review of implementation issues. Food Nutr Bull 33: S6-26. |
| 25 | Milat AJ, Bauman A and Redman S. (2015) Narrative review of models and success factors for scaling up public health interventions. Implement Sci 10: 113. |
| 26 | Lee K, van Nassau F, Grunseit A, et al. (2020) Scaling up population health interventions from decision to sustainability - a window of opportunity? A qualitative view from policy-makers. Health Res Policy Syst 18: 118. |
| 27 | Yamey G. (2012) What are the barriers to scaling up health interventions in low and middle income countries? A qualitative study of academic leaders in implementation science. Global Health 8: 11. |
| 28 | Mangham LJ and Hanson K. (2010) Scaling up in international health: what are the key issues? Health Policy Plan 25: 85-96. |
| 29 | Koorts H, Cassar S, Salmon J, et al. (2021) Mechanisms of scaling up: combining a realist perspective and systems analysis to understand successfully scaled interventions. Int J Behav Nutr Phys Act 18: 42. |
| 30 | Zomahoun HTV, Ben Charif A, Freitas A, et al. (2019) The pitfalls of scaling up evidence-based interventions in health. Glob Health Action 52 12: 1670449. |
