# Supplementary material for: From intervention studies to national programs, what are the favoring and hindering factors? a scoping review
Source: BMC Public Health. 2025 Oct 28;25:3623. doi: 10.1186/s12889-025-24770-1 (PMC12560514; doi:10.1186/s12889-025-24770-1)
Supplement: Supplementary file 5 — Additional file 5: Glossary of terms [file 12889_2025_24770_MOESM5_ESM.docx]

**Additional file 5. Glossary of terms.**

| **Term** | **Definition** |
| --- | --- |
| Dissemination | Group of activities focused on the distribution of information to a specific group or clinical practice (6) |
| Dissemination research | Scientific study of the phenomena to understand how to better spread knowledge (6) |
| Implementation | Integration of evidence-based health interventions into community settings, focusing on increase positive outcomes in health (6) |
| Implementation science | It is the “research that empirically examines and tests the translation of effective programs in new settings and populations (replication) and on a larger scale in whole populations (dissemination/ scale-up)." Considering the focus of this project, it was considered exclusively the scale-up process (2) |
| Translation | It is the process of transforming observations made in the laboratory, clinic, and community into interventions that enhance the health of both individuals and the public. This includes everything from diagnostics and treatments to medical procedures and behavioral modifications (1) |
| Translation science | Research focused on improve the effectiveness and efficiency of moving from one research stage to the next, seeking to identify the scientific and operational principles that are fundamental to each step of the translational process (1) |
| Scaling-up | After the first step of implementation science (replication), if the innovation presented good results in one specific setting, it may be interesting to expand it in this specific setting to increase the positive impact by benefiting more people (6) |
