# Supplementary material for: From intervention studies to national programs, what are the favoring and hindering factors? a scoping review
Source: BMC Public Health. 2025 Oct 28;25:3623. doi: 10.1186/s12889-025-24770-1 (PMC12560514; doi:10.1186/s12889-025-24770-1)
Supplement: Supplementary file 6 — Additional file 6: List of enabling and hindering factors [file 12889_2025_24770_MOESM6_ESM.docx]

**Additional file 6. List of enabling and hindering factors.**

| **Hindering factor** | **Number of articles** |
| --- | --- |
| ***The innovation*** | **6** |
| Multicomponent and high complexity | 3 |
| Difficult to integrate in the current supply chain | 1 |
| Reallocation of HCP | 1 |
| Not attractive to implementer teams | 1 |
| ***User organization*** | **20** |
| Absence or change of supervision | 7 |
| Problematic communication among different stakeholders | 2 |
| Not context-specific | 2 |
| Lack of adjusting implementation based on pilot findings | 1 |
| Poor/none division of responsibility | 1 |
| Poor/ none capacity of management | 1 |
| Lack/ problematic partnerships | 1 |
| Poor/none division of responsibility | 1 |
| Reduced/none HCP support | 1 |
| Poor/ none capacity of management | 1 |
| Poor/ none capacity of manage | 1 |
| Poor integration with existing staff | 1 |
| ***Environment*** | **57** |
| Administrative hurdles | 10 |
| HCP problematic conditions of working | 8 |
| Political issues | 7 |
| Lack of adequate human resources | 7 |
| Community resistance, due to moral/ prejudice/ reduced awareness | 5 |
| Reduced/ None community support or engagement | 4 |
| Limited understanding about the innovation | 4 |
| Reduced availability of materials | 3 |
| Difference of service delivery across the different states/ Local inequalities | 5 |
| Target group difficult to reach | 1 |
| Inflation and financial constraints | 1 |
| Competition with other programs | 1 |
| Reduced/ None community support or engagement | 1 |
| ***Resource team*** | **2** |
| Lack of innovative research designs | 1 |
| Lack of robust research methods | 1 |
| ***Dissemination and advocacy*** | **10** |
| Failure to actively spread knowledge of an innovation | 5 |
| Poor training | 5 |
| ***Organizational choices*** | **5** |
| Absence of scaling-up plan | 1 |
| Issues with the supply chain | 1 |
| Not involvement of relevant stakeholder(s) | 1 |
| Precipitancy scale-up pace | 1 |
| Top-down implementation | 1 |
| ***Costs/resources allocation*** | **11** |
| Insufficient resource | 8 |
| Donor driven decision-making | 1 |
| Lack of awareness about cost-effectiveness to real-world implementation | 1 |
| Absence of transparency about resource allocation | 1 |
| ***Monitoring and evaluation*** | **7** |
| Poor/none monitoring and evaluation activities | 7 |
| ***Other factors*** | **5** |
| Difficult to reach informed consent in large scale | 1 |
| Maintaining patient-centeredness | 1 |
| Negative users/ population perception about the program | 1 |
| Non-transferability/ scalability from trial conditions | 1 |
| Prohibitive costs of out-of-pocket payments for medication needed | 1 |
| **Favoring factor** | **Number of articles** |
| ***The innovation*** | **16** |
| Relevant | 3 |
| Adaptability | 3 |
| Easy to apply | 3 |
| Evidence based | 3 |
| Clarity about the target population | 1 |
| Stability | 1 |
| Interoperability | 1 |
| Extensibility | 1 |
| ***User organization*** | **33** |
| Defined strong leadership and other roles | 8 |
| Strong coordination | 8 |
| Multisectoral approach/ Partnerships | 5 |
| Commitment to improve | 3 |
| Institution implementation capacity and technical innovation | 3 |
| Accountability structures | 1 |
| User organization credibility | 1 |
| Health needs perceived | 1 |
| Integration with other implemented health program | 1 |
| Country ownership | 1 |
| Strong communication among stakeholders | 1 |
| ***Environment*** | **42** |
| Positive political environment | 11 |
| Community support and engagement | 10 |
| Positive administrative conditions | 5 |
| Community/ population awareness | 4 |
| Strong HCP engagement | 2 |
| Partnerships | 2 |
| Favoring HCP working conditions | 2 |
| Positive social environment | 1 |
| Adequate human resources | 1 |
| Charismatic leader | 1 |
| Sufficient human resource | 1 |
| Focused tasks | 1 |
| Integrated with other established and correlated services | 1 |
| ***Resource team*** | **9** |
| High level commitment | 3 |
| Simplification of elements | 2 |
| Flexible scale-up | 1 |
| Professional capacity | 1 |
| Development of quality guidelines | 1 |
| Implementation at the local level | 1 |
| ***Dissemination and advocacy*** | **26** |
| Training | 9 |
| Strong advocacy | 5 |
| Target communication | 5 |
| Mass media utilization | 3 |
| Lobbying to foster a favoring opinion | 2 |
| Data-informed advocacy | 1 |
| Awareness campaigns | 1 |
| ***Organizational choices*** | **16** |
| Well defined strategy | 4 |
| Intervention tailored to the context | 4 |
| Appropriate delivery system | 3 |
| Midcourse corrections | 2 |
| HCP recruitment in the community | 1 |
| Appropriate channels and number required to coverage | 1 |
| Strong communication strategies | 1 |
| ***Cost/ Resources mobilization*** | **12** |
| Adequate funding | 7 |
| Cost assessment | 1 |
| Cross-funding | 1 |
| External funding | 1 |
| Lobbying for funding | 1 |
| Self-financing/ stable funding | 1 |
| ***Monitoring and evaluation*** | **16** |
| Executing monitoring and evaluation activities | 10 |
| Well-defined performance metrics | 2 |
| Effective evaluation | 2 |
| Data analysis to continuous improvement | 1 |
| Utilization of different sources of data | 1 |
| ***Other factors*** | **5** |
| Community needs assessment | 1 |
| Focus on community delivery | 1 |
| Pilot studies | 1 |
| Technical consensus | 1 |
| Undertaking co-design and planning | 1 |
